# Investigating food allergy awareness and attitudes among teachers in primary schools: current status and opportunities for enhancement

**DOI:** 10.3389/fped.2024.1471494

**Published:** 2025-01-08

**Authors:** Amal H. Aljohani, Abeer Mutair Alshamani, Afnan Ahmed Aljohani, Aisha Saad Almohammadi, Bushra Saad Alharbi, Deena Faraj Altarjami, Reyouf Samer Alsaedi

**Affiliations:** Pediatric Department, College of Medicine, Taibah University, Madinah, Saudi Arabia

**Keywords:** food allergy, children, school teachers, attitude, anaphylaxis

## Abstract

**Background:**

Food allergies are common among children worldwide. This occurs when the immune system has an abnormal reaction to certain foods. This reaction can range in severity from mild to severe and may lead to anaphylaxis, which can be fatal. As teachers are the first responders in managing such situations in schools, this study aims to explore and measure primary school teachers' knowledge and attitudes about food allergies and their management and review the current school policy.

**Methods:**

This cross-sectional study uses a randomly distributed online questionnaire to measure teachers' knowledge and attitudes at primary schools in Medina, Saudi Arabia.

**Results:**

The sample consisted of 383 primary school teachers. Approximately 87.5% of the participants were aware of food allergies. More than two-thirds of teachers were able to identify anaphylactic shock. Only 15.4% of the teachers knew that an epinephrine autoinjector was the first aid measurement to be taken. Additionally, 39.9% of the teachers reported that the school does not have an action plan for dealing with students with food allergies. Approximately 93.4% of the participants agreed that avoiding foods that cause allergies is important for preventing allergies.

**Conclusions:**

This study revealed teachers' good knowledge in the identification of food allergy and their symptoms, as well as the awareness of their potential severity. However, the study highlighted a significant gap in management. Most had positive attitudes toward learning about food allergies. Therefore, administration policies and strategies need to be implemented to assist in managing food allergies at school.

## 1 Introduction

Food allergies (FAs) are a common, significant public health concern, particularly given the increasing prevalence among children. FAs are a growing global public health issue impacting children and adults (1). The prevalence of food allergies varies across different regions, with estimates suggesting that they affect approximately 10% of children (1). One study in Saudi Arabia about Food allergies reported by parents accounted for 15.2% of the total cases, but there was significant regional variation in this prevalence. The prevalence of food allergies was highest in the western region at 20.5%, followed by the central region at 16.6%. In the eastern region, the prevalence was 13.6%, while the northern and southern regions reported lower rates at 12.3% and 11.7%, respectively (2). Determining the true prevalence of food allergies is challenging due to several factors, such as a small sample size, a focus on specific populations rather than children, variations in protocols and definitions of food allergies, and the evaluation of only a limited number of food allergens (3).

Food allergy occur when the immune system reacts to certain foods. The severity of symptoms can range from mild to severe and, in rare cases, can be life-threatening. It can cause various symptoms and signs, affecting various body systems, such as the skin, gastrointestinal (GI) tract, respiratory tract, and cardiovascular systems. Skin reactions, such as acute urticaria, angioedema, and erythema, are the most common clinical manifestations of food allergies (4). Anaphylaxis is the most severe form of an allergic reaction that can be fatal (5). Food-induced anaphylaxis is the most common cause of anaphylactic reactions among children (6). In addition to the clinical implications, there is strong evidence that FAs have an impact on psychological distress, including anxiety, and the quality of life of children and adolescents (7).

In schools, approximately one in five children diagnosed with food allergies experience allergic reactions triggered by the consumption of specific foods (1). Schools play crucial roles in protecting children with food allergies, as they spend a significant part of their day there, and teachers are the first responders in managing such situations (7, 8). They must recognize and effectively manage allergic reactions to preserve children's lives. Therefore, assessing the knowledge and actions of teachers regarding children with food allergies can help effectively manage food allergies within the school setting and provide valuable insights for developing future policies related to FA education, prevention, and treatment in schools (9). Furthermore, it assists in implementing strategies aimed at minimizing the risk of allergen ingestion and ensuring prompt recognition and treatment of any allergic reactions or anaphylaxis that may occur (10). This can ultimately enhance the safety of the school environment for children with food allergies. For this reason, this study aimed to explore and measure teachers' understanding of and attitudes toward food allergies and their management.

## 2 Research methodology

This cross-sectional study was conducted with primary school teachers via an online questionnaire-based data collection distributed to 593 primary schools (private and government) in Medina City, Saudi Arabia. The school's teachers were chosen randomly from June 11 to July 17, 2023. The sample size was determined based on the total number of teachers in Madina city (9,883). The calculated sample size was 370 to obtain results at a 95% confidence interval, a margin error of 5%, and a population variance of 50%. The sample included male and female teachers at primary schools in Medina city, excluding teachers in schools outside the city.

The questionnaire's structure was developed following previous studies; some questions were modified, and new ones were added (9, 11, 12). The questionnaire comprises 39 questions divided into four main domains: sociodemographic data, knowledge, experience, and perspectives and beliefs about FAs. The sociodemographic domain included years of teaching experience, type of school, and level of education in addition to other questions. The knowledge section consisted of three parts: the definition of an FA and its symptoms, severity, and management. The experience section explores teachers' personal encounters with food allergies in school settings, how they identify students with food allergies and their awareness of the number of affected students in their school. Additionally, the perspectives and beliefs section included prevention measures, the difficulties that students with FAs encounter at school, and raising awareness of FA management.

A pilot study was performed for this research by giving the questionnaire to 37 primary school teachers (10% of the sample size); the schools were randomly selected from the distinct educational sectors to evaluate the questionnaire's validity and clarity of language and structure. It was validated and adopted as follows: firstly, the questionnaire was sent to 3 academic experts knowledgeable in this field. After coordination and consensus of all the experts' opinions, the final questionnaire was drafted, and it underwent pilot testing with 37 individuals to confirm its reliability. The stability of the questionnaire items was calculated using Cronbach's alpha coefficient. The degree of consistency between the main parts of the survey was assessed via Cronbach's alpha, illustrating an overall alpha coefficient distribution with values between 0.761 and 0.821; thus, the items used in the study were reliable and consistent. Factor analysis was performed via confirmatory factor analysis. The data-gathering method was reviewed and modified based on the feedback given during the pilot and the statistical analysis results.

The questionnaire was sent as a self-administered online survey via Monkey Survey in Arabic and English to primary school teacher groups on WhatsApp as a link with an explanation of the purpose of the study. Since all the respondents answered every question completely, there were no incomplete responses.

### 2.1 Data analysis

The Social Science Software Statistical Package, version 26.0 (SPSS Inc., Chicago, IL), was used for data analysis. Descriptive analyses were conducted to calculate the frequencies and percentages of categorical variables. The chi-square test and Fisher's exact test were used to determine if there was a significant association between teachers' knowledge and attitudes about FA and their sociodemographic variables. A *p* value of less than 0.05 was considered statistically significant, and the confidence interval was 95%.

### 2.2 Ethical considerations

All respondents in this study were asked for informed consent to participate. Ethical approval (STU-22-013) was given to this study by the Institutional Review Board (IRB) of Taibah University, College of Medicine, Research Ethics Committee (CM-RREC).

## 3 Results

The total number of responses received was 383, which exceeds the calculated sample size of 370. Although the response rate is relatively low, the sample size was sufficient for statistical analysis.

Most of whom were Saudis (95.6%). Table 1 shows the sociodemographic characteristics of the sample: 62.7% of the participants were female, and 49.1% were between 41 and 50 years old. Additionally, approximately 82% of the participants were married and had children. Most teachers (81.2%) had a bachelor's degree and worked in public schools, and half had experience equivalent to 16 years or more.

**Table 1:** Sociodemographic characteristics of the sample.

Variable	*N* (%)
Gender	
Male	143 (37.3%)
Female	240 (62.7%)
Age	
30 or less	36 (9.4%)
31–40 years	98 (25.6%)
41–50 years	188 (49.1%)
51 or more	61 (15.9%)
Nationality	
Saudi	366 (95.6%)
Non-Saudi	17 (4.4%)
Marital Status	
Single	38 (9.9%)
Married	314 (82.0%)
Divorced	22 (5.7%)
Widower	9 (2.3%)
I have children	
No	34 (8.9%)
Yes	334 (87.2%)
Education level	
Diploma	32 (8.4%)
Bachelor	313 (81.7%)
Master	34 (8.9%)
PhD	4 (1.0%)
Type of school	
Public school	307 (80.2%)
Private school	53 (13.8%)
International school	23 (6.0%)
Years of experience	
Less than 1 year	10 (2.6%)
1 to 5 years	41 (10.7%)
6 to 10 years	32 (8.4%)
11 to 15 years	95 (24.8%)
16 years and more	205 (53.5%)

Regarding the teachers' general knowledge of food allergies (Table 2), approximately 87.5% of the participants were aware of food allergies. Approximately 88.8% and 80.7% correctly identified the most common food that can cause an allergic reaction and common symptoms, respectively. However, only 15% of them could differentiate between lactose intolerance and a milk allergy. Approximately half of the teachers who knew about food allergies reported that the source of their information is either their own food allergy or that of a family member, followed by social media (Twitter, Instagram, Snapchat, etc.) (32.9%), then the internet search (Google, etc.). There was a statistically significant association between knowledge about the most common symptoms of FAs and teachers who have children (*p* = 0.032). There was no difference in knowledge between genders.

**Table 2:** Knowledge about food allergy and its symptoms.

Item	*N* (%)
Familiarity with food allergies	
No	48 (12.5%)
Yes	335 (87.5%)
Foods most likely to cause a food allergy	
Tomatoes, cucumbers, oranges, apples, spinach	6 (1.6%)
Milk, eggs, fish, shrimp, nuts, wheat, sesame	340 (88.8%)
Legumes, rice, potatoes, spices, carrots, bananas	15 (3.9%)
I do not know	22 (5.7%)
The most common symptoms of a food allergy	
Constipation, headache, nausea, fever	14 (3.7%)
Abdominal bloating, diarrhea, stomachache	47 (12.3%)
Skin rash, facial swelling, wheezing	309 (80.7%)
I do not know	13 (3.4%)
Suffering from lactose intolerance (the inability to digest dairy products fully) such as having a milk allergy	
No	58 (15.1%)
Yes	137 (35.8%)
I do not know	188 (49.1%)

In reference to FA severity, more than two-thirds of teachers were able to identify anaphylactic shock symptoms and signs, and approximately half believe that anaphylactic shock can cause death and that allergy symptoms can develop solely from contact with the allergy-inducing food (Table 3). Despite their knowledge about food allergies, the management information revealed that approximately one-third of the teachers reported that the first aid measures that must be taken in cases of severe food allergies at school were calling the ambulance service (36.8%), followed by calling the students' parents. In contrast, only 15.4% of teachers knew that an epinephrine autoinjector (EpiPen) was the first line of medication to manage anaphylaxis, and more than half of them did not know how to use it. With respect to the availability of an action plan at school, 39.9% of teachers report that the school does not have an action plan for dealing with students with food allergies, and 38.1% did not know if there was an action plan. Nevertheless, 95.6% agree that a treatment plan is needed to address food allergies at school. Teachers' overall knowledge of food allergies was assessed by summing scores from relevant questions, with the sample divided into two groups using the median as a cutoff. The results revealed that 83.8% of teachers (321 teachers) have a good knowledge.

**Table 3:** Knowledge about the severity of food allergy.

Item	*N* (%)
The most common symptoms of a severe food allergy (anaphylactic shock)	
Nausea, fever, sore throat, cough	34 (8.9%)
Red eye, runny nose, headache	15 (3.9%)
Skin rash, swelling in the face, throat tightness, loss of consciousness	297 (77.5%)
I do not know	37 (9.7%)
The belief is that people with food allergies may develop allergy symptoms after touching the food that causes them.	
No	109 (28.5%)
Yes	188 (49.1%)
I do not know	86 (22.5%)
The belief that a severe food allergy (anaphylactic shock) can cause death	
No	62 (16.2%)
Yes	187 (48.8%)
I do not know	134 (35.0%)
First aid measures that must be taken in the case of a severe food allergy (anaphylactic shock) at school	
Perform CPR	9 (2.3%)
Give antihistamines	43 (11.2%)
Tell the student's family to take him to the hospital	104 (27.2%)
Call the ambulance service	141 (36.8%)
Give an epinephrine injection(self-injection)	26 (6.8%)
I don't know	60 (15.7%)
Belief that self-injecting epinephrine (EpiPen) is the first line of medication for the management of anaphylaxis	
No	47 (12.3%)
Yes	59 (15.4%)
I do not know	277 (72.3%)
Knows how to use an autoinjector (EpiPen)	
No	35 (59.3%)
Yes	24 (40.7%)
The school has an action plan for dealing with students with food allergies.	
No	153 (39.9%)
Yes	84 (21.9%)
I do not know	146 (38.1%)
I have information about the action plan.	
No	37 (44%)
Yes	47 (56%)
A treatment plan is needed to deal with food allergies at school.	
Strongly agree	283 (73.9%)
Agree	83 (21.7%)
Neutral	15 (3.9%)
Disagree	0 (0%)
Strongly disagree	2 (0.5%)

With respect to their experience with FA, Table 4 shows that approximately 45% of teachers stated that they had encountered a student with a food allergy (FA) during their work as teachers. Around two-thirds of them knew about the allergy from the student's parent or the students themselves. In comparison, 11.8% knew from doctor's medical reports, and 10% of the allergic symptoms appeared in the students at school. Additionally, 70.8% of teachers reported that they do not know how many students have FA in their school. In addition, there was a statistically significant association between knowledge about the severity of FA and teachers who have experience with students who have food allergies, they have better knowledge about the severity of symptoms than teachers who do not have students with food allergies (*p* = 0.021).

**Table 4:** Teachers' experience regarding food allergy.

Item	*N* (%)
I have had students who have had food allergies during my work as a teacher	
No	213 (55.6%)
Yes	170 (44.4%)
If yes, how did you know about his food allergy?	
A report from a doctor to the school	20 (11.8%)
An emergency card carried by the student	2 (1.2%)
Have been told by the parents	98 (57.6%)
The student themself	30 (17.6%)
Allergy symptoms appeared in the student	17 (10.0%)
How many students have food allergies in your school?	
None	33 (8.6%)
1	15 (3.9%)
2–5	36 (9.4%)
>5	28 (7.3%)
I do not know	271 (70.8%)

Approximately 93.4% of the participants agreed that avoidance of the food that causes the allergy is an important step to prevent the allergy. Additionally, 60.1% believe that a canteen free of food allergens is the way to avoid FA attacks from occurring in schools by banning allergenic foods at school (44.9%), avoiding food of unknown content (39.4%), and not allowing meals to be shared among students (35.5%). Most of the teachers (82.8%) reported that they ensure that packaged food is “safe” from allergens by reading food labels, and they believed that there is a need to supervise students with food allergies while eating (Supplementary Table S5). For further analysis, prevention measures at school were stratified according to whether the teachers had students with food allergies. This analysis revealed that teachers who have had a student with a food allergy compared with those who have not are more likely to agree that there is a need to supervise students who suffer from FAs while eating (*p* < 0.002).

Regarding the perspectives of teachers on the difficulties faced by students with food allergies at school: 32.9% thought that students with food allergies suffer from psychological problems, followed by social relationship difficulties (15.1%) and academic difficulties (2.3%). In comparison, 50% of teachers believe they do not have any difficulties. Almost two-thirds of the participants agreed that it is not feasible to stay away from the food that he or she is allergic to, and 55.1% of them feel responsible for a student who suffers from a FA (Supplementary Table S6). However, teachers believe that the main challenges in managing food allergies at school included communicating with parents and knowing the student with a FA (44.6%), the absence of a specialized medical team or nurse (43.9%), and a lack of awareness about food allergies and dealing with them (43.6%). Additionally, 74.7% of teachers who had students with food allergies felt responsible for them (*p* < 0.001 39.4% vs. 74.7%).

With respect to managing food allergies at school, approximately 50% of participants believed that food allergies and anaphylaxis can be handled by school staff (Supplementary Table S7). In addition, 96.1% of them agreed that there is a need to raise awareness about food allergies in the school through periodic training of the staff (42.3%), lectures in the school (30.8%), workshops (10.7%), social media (7.3%), books/leaflets (4.4%), and online lectures (3.4%). Additionally, there was a statistically significant association between teachers who believe there is a need to raise awareness about food allergies at school, and teachers who had students with FAs (*p* = 0.006) (74.1% vs. 58.2%).

## 4 Discussion

To assess the overall understanding of allergies, knowledge was divided into three sections: knowledge about food allergy and its symptoms, knowledge about the severity of food allergies, and knowledge about the management of food allergies. By categorizing the knowledge into these three domains, the depth of teachers' understanding in each area can be evaluated separately. This approach allows for a more detailed assessment of their knowledge and helps identify specific areas where further education may be needed.

This research revealed insufficient knowledge regarding food allergy management among teachers. Although the majority of respondents could accurately identify common foods responsible for food allergies and teachers who have students with food allergies, exhibit a good level of awareness of food allergies and their associated symptoms, only a few participants could correctly identify the appropriate first aid measure for anaphylactic shock. In this study, primary school teachers have a good level of awareness of food allergy symptoms (83.8%), which is higher than what was reported in previous studies, which reported rates of 16%, 52.2%, 69.7%, and 70.8% (8, 11–13). In Alzahrani et al., only 14.5% of participants exhibited a high level of awareness, scoring above 60% on the knowledge questionnaire regarding food allergies (14).

Approximately 50.2% of teachers who were aware of FAs reported that the source of their information was either that they had a FA or that a family member had a FA, followed by an internet search (Google, etc.) (32.9%), then social media (Twitter, Instagram, Snapchat, etc.) (32.6%) (Supplementary Figure S1). This result may be explained by the high use of the internet and social media in this area. The widespread availability of internet-based learning applications facilitating distance learning presents an appropriate opportunity for cost-effective training (13).

We observed that a few teachers (15.1%) could differentiate between a milk allergy and lactose intolerance. This percentage is significantly lower than the findings of Kanter et al. (63%) (11). This study, in line with the results of Alomran H et al., demonstrates that most participants identified skin rashes, facial swelling, and wheezing as the most common symptoms of food allergies (13). Additionally, most participants (88.8%) correctly recognized the most common foods that trigger food allergies (Table 2). Fortunately, many food allergies in children, such as those to milk, eggs, and wheat, tend to resolve as they grow older. In contrast, allergies to peanuts, tree nuts, and fish may persist for a lifetime (3). In addition, approximately half of the participants believed that individuals with food allergies may develop symptoms of a FA even by touching the food and recognized the possibility of death resulting from severe food allergies (Table 3) (15). This percentage is higher than what was reported in the previous studies (13). In regard to dealing with FA, around one-third of the participants thought that the first aid measure for anaphylactic shock at school is to contact emergency services. In comparison, 27.2% of teachers believed it is preferable to inform the student's family (Table 3). These responses also relate to the current administrative policy regarding managing school emergencies, including severe allergic reactions. Additionally, there is a low level of awareness regarding the administration of epinephrine injections (self-injections) (6.8%), as the first-line treatment for anaphylaxis at school; these results are comparable to previously reported studies, which reported values of 13.3%, 12.2%, and 0% (12, 13, 16). However, of the teachers who were aware of epinephrine injections, approximately 40% knew how to administer them, which is a high percentage compared to previous studies, which reported values of 26.7% and 6.8% (Table 3) (12, 13). Regarding teachers who were aware of action plans for dealing with students with food allergies in school, they represented 21.9% of participants, which is comparable to that reported in previous studies19.2%, 8.2%, and 5.9% (12, 13, 17). Furthermore, almost all teachers agreed that schools should have a management plan to address children who have food allergies safely (Table 3). This highlights their understanding of the necessity of a management plan and their willingness to have it and be involved in the management of their students (9).

In relation to experience, the findings indicate that 44.4% of teachers have had students with food allergies, which is lower than the percentage reported in another study (16). However, the prevalence of parent-reported FAs was only 15%. This could reflect a misperception of FA prevalence among teachers or a lack of communication between parents and teachers about food allergies (2). Approximately 70.8% of the teachers did not know if their school had students with food allergies, which may reflect inappropriate communication inside the school regarding such health problems (Table 4).

This study shows that teachers who have children had a higher level of awareness about the most common symptoms of FA (80.2%) compared with teachers who do not have children (*p* = 0.032). In addition, we find that half of the participants were aged 41–50 years, and 87.2% of them had children, which may contribute to a high level of awareness of FAs. This result was in agreement with L. Alzahrani's study, which reported the same findings (14).

Approximately 65.5% of teachers know the importance of avoiding allergenic foods as preventive measures for allergies and preventing accidental exposure to allergic foods, including having allergen-free canteens. Additionally, half of them support a complete ban on allergenic foods and emphasized restricting foods with unknown origins, whereas 35.5% believed preventing meal sharing among students could reduce the degree of risk, and 13.6% expressed the opinion that prevention might be challenging. Similar to prior studies, most teachers believe that avoidance is the most effective method for preventing allergies (Supplementary Table S5) (8, 13).

Children with food allergies face a variety of social difficulties, such as teasing, bullying, and discomfort from unwanted attention; all of these issues affect their quality of life (18). Additionally, they felt isolated because of school rules that physically kept them apart from other children, such as designating (19). In this study, we assessed teachers' opinions about the main difficulties faced by students with food allergies. Approximately 32.9% agreed that the students faced psychological difficulties, 15.1% agreed that they faced difficulties in social relationships, and only 2.3% agreed that students with FAs have academic difficulties (Supplementary Table S6). These results reflect the underestimation of psychological problems that students with food allergies may encounter at school. This result is in line with those obtained by L. Polloni et al., which were emotional consequences (37.2%), social difficulties (10.2%), and learning difficulties (4.3%) (9). A study done by Takrouni et al. showed that 27.7% of the participants felt that children with food allergies face bullying at school because of their allergies (20). Although avoidance is the first step in managing FAs, we found that 73.4% reported that it is difficult to stay away from the food the student is allergic to, which is higher than the 33.7% reported by Madooh et al. (Supplementary Table S6) (8). Interestingly, most participants thought that they could manage FA and anaphylaxis at school, demonstrating a proactive attitude and preparedness to improve.

The majority of children with food allergies experience allergic reactions, even in the most careful and informed environments (21). Since the majority of people who have died from FA reactions were outside of their homes, it would seem that their concerns regarding activities outside of the house are well-founded (11). Most of the participants agreed that there is a need to raise awareness about food allergies in the school through periodic training of the staff and lectures in the school (Supplementary Table S7). Educational sessions can play an excellent role in improving the teachers' knowledge and practice toward anaphylaxis (11).

This study revealed that teachers generally have a good understanding of food allergy symptoms and severity, mostly gained through personal experience or self-education. However, there is a lack of knowledge about managing and properly handling students with allergic reactions at school. This study also points out the absence of organized, regular education and, more importantly, a school policy and guidelines for managing food allergies. There are no specific guidelines for food allergy management in primary schools.

To create a suitable management plan for each student, the reported guidelines emphasize a coordinated approach involving the parents of children with food allergies, school staff, and medical providers and the need for multidisciplinary collaboration and efficient communication between all (22, 23). We think policy-makers in the ministries of education and health need to be involved in implementing policies and guidelines regarding school staff education and FA management and periodically evaluating them.

The study has a few noteworthy limitations. The study setting was focused on one city only (Medina city). In general, more reliable results could be obtained if we included other regions. Self-selection bias may have occurred. The female responses were more than the male, and the responses from government schools were more than those from private schools.

## 5 Conclusion

In conclusion, this study revealed that teachers' good food allergy knowledge in identifying food allergies and their symptoms, as well as the awareness of their potential severity. However, the study highlighted a significant gap in food allergy management. Most had positive attitudes toward learning about food allergies. Additionally, it is crucial to implement policies and guidelines to help schools deal with students with food allergies.

## Data Availability

The original contributions presented in the study are included in the article/Supplementary Material, further inquiries can be directed to the corresponding author.
