# Supplementary material for: Investigating food allergy awareness and attitudes among teachers in primary schools: current status and opportunities for enhancement
Source: Front Pediatr. 2025 Jan 8;12:1471494. doi: 10.3389/fped.2024.1471494 (PMC11750800; doi:10.3389/fped.2024.1471494)
Supplement: Supplementary file 1 [file Table1.docx]

Table 5: Prevention measures at school

| N (%) | **Item** |
| --- | --- |
| **Avoiding the food that causes allergy is an important step to prevent the allergy.** | |
| 251 (65.5%) | Strongly agree |
| 107 (27.9%) | Agree |
| 22 (5.7%) | Neutral |
| 3 (0.8%) | Disagree |
| 0 (0%) | Strongly disagree |
| **Food allergy attacks can be prevented in your school by: (You can select all that apply)** | |
| 52 (13.6%) | It is not possible |
| 136 (35.5%) | No meal sharing among students |
| 230 (60.1%) | The canteen is free of common allergenic foods |
| 151 (39.4%) | Avoiding food of unknown content |
| 172 (44.9%) | Banning allergenic foods at school |
| **How can you ensure packaged food is “safe” from a specific allergen? (You can choose all that apply)** | |
| 82 (21.4%) | Ask someone |
| 18 (4.7%) | Tasting the food |
| 317 (82.8%) | Reading food labels |
| 23 (6.0%) | It is not possible |
| 39 (10.2%) | I don’t know |
| **There is a need to supervise students who suffer from food allergy while eating.** | |
| 184 (48.0%) | Strongly agree |
| 136 (35.5%) | Agree |
| 58 (15.1%) | Neutral |
| 4 (1.0%) | Disagree |
| 1 (0.3%) | Strongly disagree |

Table 6: Difficulties faced by students with food allergies at school

| N (%) | **Item** |
| --- | --- |
| **The main difficulties faced by students with food allergy** | |
| 58 (15.1%) | Difficulties in social relationships |
| 9 (2.3%) | Academic difficulties |
| 126 (32.9%) | Psychological difficulties |
| 190 (49.6%) | Do not have any social, academic, or psychological difficulties |
| **For someone who has a food allergy, it is difficult to stay away from the food that they are allergic to.** | |
| 73 (19.1%) | Strongly agree |
| 208 (54.3%) | Agree |
| 73 (19.1%) | Neutral |
| 25 (6.5%) | Disagree |
| 4 (1.0%) | Strongly disagree |
| **Children with food allergies have overprotective parents.** | |
| 67 (17.5%) | Strongly agree |
| 174 (45.4%) | Agree |
| 113 (29.5%) | Neutral |
| 27 (7.0%) | Disagree |
| 2 (0.5%) | Strongly disagree |
| **How do you feel about a student who suffers from a food allergy at school?** | |
| 44 (11.5%) | Anxiety and fear |
| 9 (2.3%) | Helplessness |
| 211 (55.1%) | Responsibility |
| 5 (1.3%) | I don’t feel anything |
| 114 (29.8%) | I have never had a student with a food allergy |
| **The main difficulties in managing food allergy and anaphylaxis at school (can choose all that apply)** | |
| 171 (44.6%) | Communication with the parents and knowing the student with a food allergy |
| 167 (43.6%) | Lack of awareness about the food allergy and dealing with it |
| 100 (26.1%) | Lack of necessary medications |
| 168 (43.9%) | The absence of a specialized medical team or a nurse |
| 86 (22.5%) | Difficulty in providing allergen-free food |
| 92 (24.0%) | Difficulty in supervising the students |
| 28 (7.3%) | I don’t know |

Table 7: Raising awareness of food allergy management at the school

| N (%) | **Item** |
| --- | --- |
| **Food allergy and anaphylaxis could be managed at school by school personnel.** | |
| 64 (16.7%) | Strongly agree |
| 126 (32.9%) | Agree |
| 128 (33.4%) | Neutral |
| 45 (11.7%) | Disagree |
| 20 (5.2%) | Strongly disagree |
| **There is a need to raise awareness about food allergies at school.** | |
| 250 (65.3%) | Strongly agree |
| 118 (30.8%) | Agree |
| 14 (3.7%) | Neutral |
| 1(0.3%) | Disagree |
| 0 (0%) | Strongly disagree |
| **The best way to raise food allergy awareness at school** | |
| 41 (10.7%) | Workshops |
| 162 (42.3%) | Periodic staff training at the school |
| 118 (30.8%) | Lectures in school |
| 17 (4.4%) | Books/Leaflets |
| 28 (7.3%) | Social media |
| 13 (3.4%) | Online lectures |

Table 8: Association between knowledge about food allergy and its symptoms and teachers who have children.

| Item | Categories | Children | | P-value |
| --- | --- | --- | --- | --- |
|  |  | Without | With |  |
| Familiarity with food allergies | No | 3 (8.8%) | 42 (12.6%) | .783 |
|  | Yes | 31 (91.2%) | 292 (87.4%) |  |
| Foods most likely to cause food allergy | Tomatoes, Cucumbers, Oranges, Apples, Spinach | 0 (0.0%) | 6 (1.8%) | .545 |
|  | Milk, egg, fish, shrimp, nuts, wheat, sesame | 31 (91.2%) | 295 (88.3%) |  |
|  | Legumes, rice, potatoes, spices, carrot, banana | 0 (0.0%) | 15 (4.5%) |  |
|  | I do not know | 3 (8.8%) | 18 (5.4%) |  |
| The most common symptoms of food allergy | Constipation, headache, nausea, fever | 2 (5.9%) | 12 (3.6%) | .032* |
|  | Abdominal bloating, diarrhea, Stomachache | 0 (0.0%) | 44(13.2%) |  |
|  | Skin rash, facial swelling, wheezing | 30 (88.2%) | 268(80.2%) |  |
|  | I do not know | 2 (5.9%) | 10 (3.0%) |  |
| Suffering from lactose intolerance (the inability to fully digest dairy products), such as having a milk allergy | No | 7 (20.6%) | 50 (15.0%) | .627 |
|  | Yes | 10 (29.4%) | 119 (35.6%) |  |
|  | I do not know | 17 (50.0%) | 165 (49.4%) |  |

Table 9: Association between knowledge about the severity of food allergy and teachers who had students with FA

| Item | Categories | teachers who had students with FA | | P-value |
| --- | --- | --- | --- | --- |
|  |  | No | Yes |  |
| The most common symptoms of severe food allergy (anaphylactic shock) | Nausea, fever, sore throat, cough | 20 (9.4%) | 14 (8.2%) | .025* |
|  | Red eye, runny nose, headache | 7 (3.3%) | 8 (4.7%) |  |
|  | Skin rash, swelling in the face, throat tightness, loss of consciousness | 157 (73.7%) | 140 (82.4%) |  |
|  | I do not know | 29 (13.6%) | 8 (4.7%) |  |
| The belief is that people with food allergies may develop allergy symptoms only after touching the food that causes them | No | 59 (27.7%) | 50 (29.4%) | .589 |
|  | Yes | 102 (47.9%) | 86 (50.6%) |  |
|  | I do not know | 52 (24.4%) | 34 (20.0%) |  |
| The belief that severe food allergy “anaphylactic shock” can cause death | No | 34 (16.0%) | 28 (16.5%) | .251 |
|  | Yes | 97 (45.5%) | 90 (52.9%) |  |
|  | I do not know | 82 (38.5%) | 52 (30.6%) |  |
| First aid measures must be taken in case of severe food allergy “anaphylactic shock” at school: | Do CPR | 6 (2.8%) | 3 (1.8%) | .160 |
|  | Give antihistamines | 24 (11.3%) | 19 (11.2%) |  |
|  | Tell the student’s family to take him to the hospital | 54 (25.4%) | 50 (29.4%) |  |
|  | Call ambulance service | 81 (38.0%) | 60 (35.3%) |  |
|  | Give epinephrine injection(self-injection) | 9 (4.2%) | 17 (10.0%) |  |
|  | I don’t know | 39 (18.3%) | 21 (12.4%) |  |
| Belief that self-injecting epinephrine (EpiPen) is the first line of medication for the management of anaphylaxis | No | 22 (10.3%) | 25 (14.7%) | .021* |
|  | Yes | 25 (11.7%) | 34 (20.0%) |  |
|  | I do not know | 166 (77.9%) | 111 (65.3%) |  |
| Who knows how to use an autoinjector (EpiPen) | No | 16 (64.0%) | 19 (55.9%) | .531 |
|  | Yes | 9 (36.0%) | 15 (44.1%) |  |
| The school has an action plan for dealing with students with food allergies | No | 90 (42.3%) | 63 (37.1%) | < .001* |
|  | Yes | 30 (14.1%) | 54(31.8%) |  |
|  | I do not know | 93 (43.7%) | 53 (31.2%) |  |
| I have information about the action plan | No | 15 (50.0%) | 22 (40.7%) | .413 |
|  | Yes | 15 (50.0%) | 32 (59.3%) |  |
| A treatment plan is needed to deal with food allergies at school | Strongly agree | 151 (70.9%) | 132 (77.6%) | .009* |
|  | Agree | 47 (22.1%) | 36 (21.2%) |  |
|  | Neutral | 14 (6.6%) | 1 (0.6%) |  |
|  | Strongly disagree | 1 (0.5%) | 1 (0.6%) |  |

^a^ Fisher's Exact Test. * P-value < 0.05 is statistically significant

Table 10: Association between prevention measures at school and teachers who had students with FA

| Item | Categories | teachers who had students with FA | | P-value |
| --- | --- | --- | --- | --- |
|  |  | No | Yes |  |
| Avoiding the food that causes allergy is an important step to prevent the allergy | Strongly agree | 129 (60.6%) | 122 (71.8%) | .113 |
|  | Agree | 69 (32.4%) | 38 (22.4%) |  |
|  | Neutral | 13 (6.1%) | 9 (5.3%) |  |
|  | Disagree | 2 (0.9%) | 1 (0.6%) |  |
| There is a need to supervise students who suffer from food allergy while eating | Strongly agree | 90 (42.3%) | 94 (55.3%) | .002* |
|  | Agree | 75 (35.2%) | 61 (35.9%) |  |
|  | Neutral | 43 (20.2%) | 15 (8.8%) |  |
|  | Disagree | 4 (1.9%) | 0 (0.0%) |  |
|  | Strongly disagree | 1 (0.5%) | 0 (0.0%) |  |
