# Supplementary figures and images for: Investigating food allergy awareness and attitudes among teachers in primary schools: current status and opportunities for enhancement
Source: Front Pediatr. 2025 Jan 8;12:1471494. doi: 10.3389/fped.2024.1471494 (PMC11750800; doi:10.3389/fped.2024.1471494)

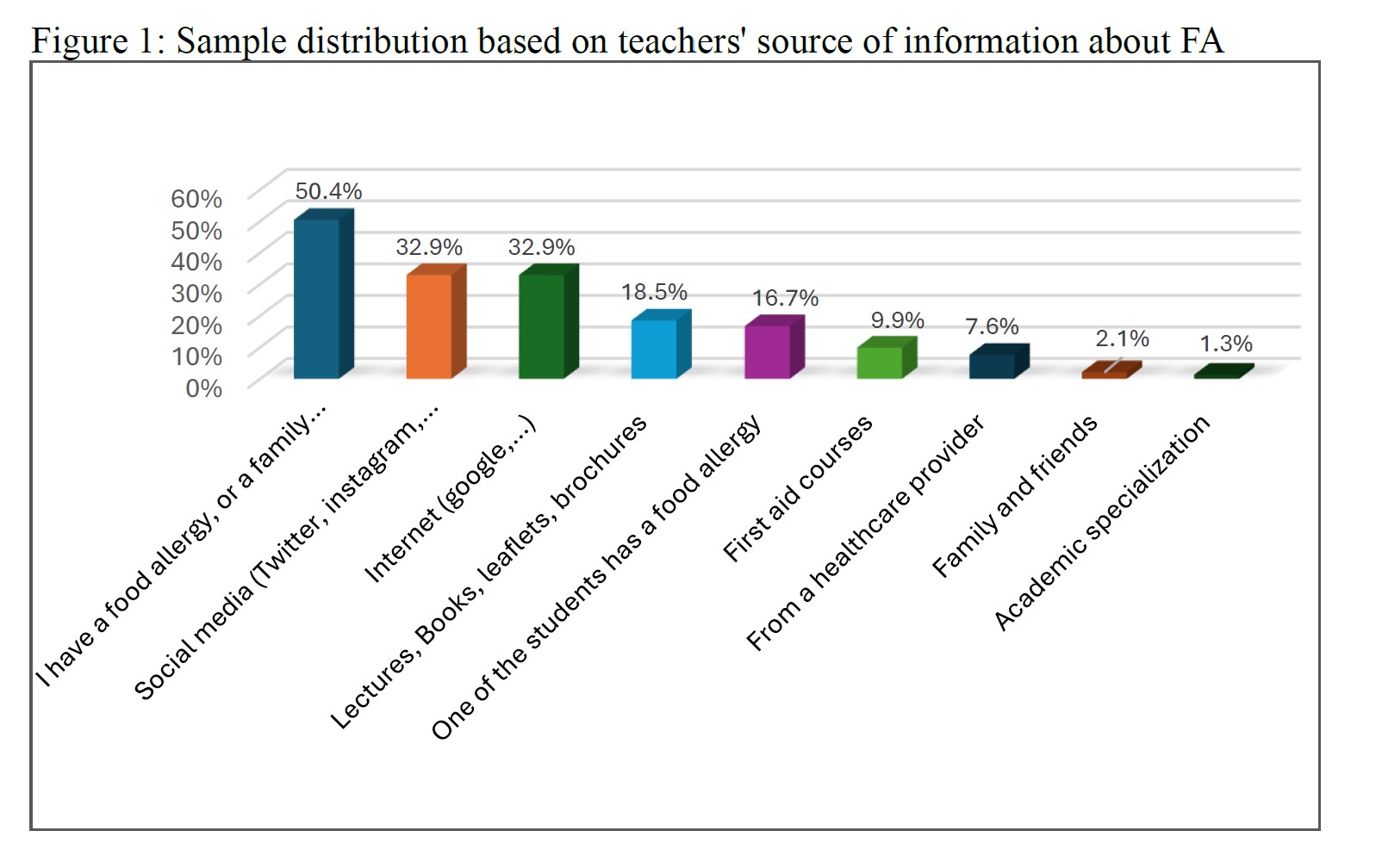

Supplement: Supplementary file 2 [file Image1.jpeg]
